# Attentional bias for high-calorie food cues by the level of hunger and satiety in individuals with binge eating behaviors

**DOI:** 10.3389/fnins.2023.1149864

**Published:** 2023-07-13

**Authors:** Ji-Min Woo, Gi-Eun Lee, Jang-Han Lee

**Affiliations:** Department of Psychology, Chung-Ang University, Seoul, Republic of Korea

**Keywords:** binge eating, hunger and satiety, food cues, incentive salience, attentional bias

## Abstract

**Introduction:**

The abnormal hyperreactivity to food cues in individuals with binge eating behaviors could be regulated by hedonic or reward-based system, overriding the homeostatic system. The aim of the present study was to investigate whether attentional bias for food cues is affected by the level of hunger, maintaining the normal homeostatic system in individuals with binge eating behaviors.

**Methods:**

A total of 116 female participants were recruited and divided into four groups: hungry-binge eating group (BE) (*n* = 29), satiated BE (*n* = 29), hungry-control (*n* = 29), satiated control (*n* = 29). While participants completed a free-viewing task on high or low-calorie food cues, visual attentional processes were recorded using an eye tracker.

**Results:**

The results revealed that BE group showed longer initial fixation duration toward high-calorie food cues in both hunger and satiety condition in the early stage, whereas the control group showed longer initial fixation duration toward high-calorie food cues only in hunger conditions. Moreover, in the late stage, the BE group stared more at the high-calorie food cue, compared to control group regardless of hunger and satiety.

**Discussion:**

The findings suggest that automatic attentional bias for food cues in individuals with binge eating behaviors occurred without purpose or awareness is not affected by the homeostatic system, while strategic attention is focused on high-calorie food. Therefore, the attentional processing of food cues in binge eating group is regulated by hedonic system rather than homeostatic system, leading to vulnerability to binge eating.

## 1. Introduction

In clinical settings, the consumption of a relatively large amount of food within a short period compared to usual, along with a subjective difficulty in controlling eating behavior during this time, is defined as binge eating behavior (American Psychiatric Association, 2013). This binge eating behavior is a core symptom observed in eating disorders such as binge eating disorder (BED), the binge-purge subtype of anorexia nervosa (AN), and bulimia nervosa (BN). Binge eating behavior often leads individuals to consume more energy than the actual amount of calories needed, resulting in imbalances related to the body and weight, causing excessive weight gain and associated psychological distress (Tanofsky-Kraff and Yanovski, 2004; Wonderlich et al., 2009). Problematic binge eating behavior can be explained by one of the important theories in addiction called the incentive-sensitization theory (Robinson and Berridge, 1993; Berridge and Robinson, 2016). The incentive-sensitization theory was proposed to explain substance abuse, such as drug addiction, and suggests that when individuals are repeatedly exposed to stimuli, such as specific substances, that provide rewarding experiences, pleasure is induced and dopamine is activated, stimulating the brain and strengthening the connection between the stimulating substance and the rewarding response of pleasure. With the reinforcement of this connection over time, individuals can become conditioned to engage in behaviors that continuously seek out and consume the rewarding stimuli. In other words, the behavior of individuals who excessively seek and consume food in binge eating and those who exhibit problematic substance addiction, characterized by excessive preoccupation and approach toward addictive substances, share the same dopamine neural pathway, indicating that cravings for specific substances or food and the triggering of such cravings may have similarities (Schulte et al., 2016; Novelle and Diéguez, 2018). In essence, similar to substance addiction, repetitive and persistent binge eating behavior is suggested to induce sensitization in the mesocorticolimbic dopamine system (Berridge, 2009). Therefore, for individuals engaging in binge eating behavior, the importance of food stimuli or cues that trigger rewarding experiences after consumption gradually increases in their daily lives or environment.

In fact, the act of human food consumption appears to rely on both a pathway that maintains physiological homeostasis and a pathway that pursues psychological satisfaction in contrast (Lutter and Nestler, 2009). For instance, the pathway aimed at maintaining physiological homeostasis is essentially a system that regulates the body's energy balance, and when energy stores are depleted, it increases appetite and motivation to seek food. On the other hand, the activation of the non-homeostatic pathway, triggered by food stimuli or cues, disregards the regulation of the system for maintaining bodily homeostasis, leading to excessive food intake, and triggering binge eating behavior, which can manifest as symptoms of overweight or obesity-related eating disorders (Berthoud, 2012; Dileone et al., 2012; Witt and Lowe, 2014; Yu et al., 2015). Particularly, individuals who engage in binge eating behavior, influenced by the hedonic system, have been found to prefer high-calorie foods and show a tendency to consume them in greater quantities compared to non-binge eating behaviors (Raymond et al., 2003). This is believed to be because high-calorie foods elicit stronger reward-related responses in the brain, leading individuals to experience a heightened sense of reward or pleasure when consuming these foods.

As mentioned earlier in the incentive-sensitization theory, food cues and rewarding experiences can be conditioned through associative and reinforcement learning processes (Berridge and Robinson, 2003). In other words, specific food cues that are consistently paired with rewarding experiences after consumption can become attractive and desired stimuli that more easily and quickly capture an individual's attention, triggering cravings. The tendency of individuals who have become sensitive to these incentive stimuli is often measured by behavioral responsiveness, such as their reaction time to specific stimuli. Particularly, the most fundamental characteristic of attention, which is the underlying process guiding individual behavior, can be more accurately and sensitively measured through attention bias (Schag et al., 2013; Popien et al., 2015). Methods such as the go/no-go paradigm (Veling et al., 2017) or the dot-probe paradigm (Fenske and Raymond, 2006; Chen et al., 2016) are useful for measuring behavioral responsiveness by observing which stimuli among various stimuli presented in the environment receive more attention or focus. However, these methods have limitations when it comes to assessing more immediate and automatic responsiveness to specific stimuli, as participants may learn during the task about certain stimuli or processes presented to them. The free-viewing paradigm using eye-tracking is an appropriate method for investigating attentional responses to food in individuals exhibiting binge eating behavior (Cisler and Koster, 2010). Enhanced attention refers to the rapid detection of salient stimuli through automatic processing in the early stages, while disengagement involves strategic processing during the maintenance of attention. Therefore, difficulty in disengagement represents sustained attention to food-related cues. Results in adults with binge eating disorder reflect longer attentional dwell time on food stimuli, indicating extended gaze duration on food cues, and eye-tracking studies have yielded mixed results regarding initial direction biases (Schag et al., 2013; Popien et al., 2015; Schmidt et al., 2016; Sperling et al., 2017). For instance, one study using the free-viewing paradigm and anti-saccade tasks found that individuals with binge eating disorder who were obese or overweight displayed longer gaze durations on food stimuli compared to individuals with obesity without binge eating disorder and normal-weight participants. However, all participants exhibited initial fixations occurring more frequently on food cues (Schag et al., 2013). Another study found that adults without binge eating disorder showed longer fixations and dwell times on both high-calorie and low-calorie food items (Popien et al., 2015). In adolescents with binge eating disorder, gaze durations were longer, but no directional biases were observed (Schmidt et al., 2016). Finally, while both positive and control groups did not differ in initial fixation locations, the positive group showed greater interest in food. There were no differences in detection times between groups in the visual search task, but the detection bias toward food cues was only found in the overall binge eating disorder (Sperling et al., 2017).

In addition, as emphasized in the incentive-sensitization theory, two key concepts are highlighted (Pool et al., 2015, 2016). First, the subjective value of incentives can vary depending on individuals' circumstances. For example, a stimulus that is rewarding to one person may be perceived as aversive or costly to another person. Second, individual circumstances and relational states are important factors that modulate sensitivity to incentives. For instance, individuals may become more sensitive to stimuli that can induce certain states they require. This is exemplified by individuals who require satiety being more sensitive to food cues or stimuli (Zhang et al., 2009; Robinson and Berridge, 2013). Evidence regarding the modulation of attentional patterns to food cues by hunger has been obtained through studies involving normal-weight and individuals with obesity (Nijs et al., 2010; Loeber et al., 2013). Normal-weight individuals showed biased attention toward food cues when hungry but not when satiated, indicating that attentional processing in healthy individuals is modulated by the homeostatic system (Piech et al., 2010; Loeber et al., 2013). However, in obese and overweight groups, no differences in attentional patterns were observed between hungry and satiated states, and in some cases, results were contrary to those of the normal-weight group (Nijs et al., 2010). The evidence considering hunger and satiety factors in individuals with binge eating is limited. Given the mixed results regarding whether hunger can trigger binge eating, it is necessary to consider both hunger and satiety factors in individuals with binge eating disorder (Stice et al., 2008).

Due to a lack of control over hunger levels, there may be mixed results in the early stages of attention, as previous studies have shown (Schag et al., 2013; Schmidt et al., 2016; Sperling et al., 2017). For instance, one study found that individuals with binge eating disorder (BED) reported significantly higher levels of hunger, more depressive symptoms, and less positive emotional responses to food cues compared to a control group (Sperling et al., 2017). Another study focusing on adolescents with BED showed that attentional biases toward food cues were only associated with increased hunger in the BED group. These results differ from previous evidence of general biases toward food stimuli in control groups, suggesting that hunger levels may have influenced the attention patterns of the control group (Schag et al., 2013; Schmidt et al., 2016). The existing evidence regarding orientation biases is not conclusive because it did not employ a competitive paradigm involving three types of stimuli in complex naturalistic scenes. Additionally, the findings regarding the early stage of attentional processes are not certain due to the relatively long duration of stimulus presentation (8 s), which may not adequately measure early covert attention (Popien et al., 2015).

In the context of the incentive-sensitization theory, incentive salience refers to the implicit motivation to obtain a reward (i.e., wanting). Therefore, it is necessary to investigate the clear results of early attentional processes, which reflect relatively automatic attention patterns (Fox et al., 2001). The brain circuitry underlying the psychological processes of the reward system consists of two components: “wanting” and “liking” (Berridge and Robinson, 2016). “Wanting” represents the motivation to obtain a reward, while “liking” refers to the pleasure experienced during consumption (Berridge, 2009). The theory suggests that “wanting” and “liking” can be independent in psychopathological conditions such as addiction or binge eating (Finlayson et al., 2007; Pool et al., 2016). Unlike “liking”, it is proposed that explicit and implicit “wanting” rely on different psychological mechanisms (Berridge and Robinson, 2003; Anselme and Robinson, 2015). Implicit “wanting” is expected to be associated with the early stage of attention, while explicit “wanting” is more closely related to overt attention, which is measured in the later stages of attention (Fox et al., 2001; Pool et al., 2016). In this study, attentional bias indicating implicit “wanting” and self-reported explicit “wanting” and “liking” was measured.

The objective of this study is to investigate the influence of hunger and satiety on visual attentional bias toward food cue images in individuals with binge eating disorder. The research hypotheses are as follows: (1) In the hunger condition, both individuals with binge eating disorder and weight-matched controls will exhibit attentional bias toward high-calorie food cues compared to both low-calorie food cues and non-food cues. (2) In the satiety condition, individuals with binge eating disorder will continue to display attentional bias, whereas the control group will show reduced attention toward high-calorie food cues.

## 2. Materials and methods

### 2.1. Participants

Prior to the experiment, candidate participants were recruited through an internet bulletin board of universities in Seoul, Korea. As an initial screening for the binge eating (BE) problem group and control group, a total of 435 female undergraduates completed the Eating Disorder Diagnostic Scale (EDDS; Stice et al., 2000) and Eating Disorder Examination Questionnaire (EDE-Q; Fairburn and Beglin, 1994). All group members with BE reported an average at least one BE episode per week for the past 3 months without compensatory behavior following BE episodes. These individuals, who have not received an official diagnosis of BED, but demonstrate relatively high scores on measures assessing symptoms of binge eating, refer to individuals with a propensity for binge eating behaviors. By contrast, none of the control group members reported BE episodes per week during the past 3 months and a history of other eating disorder symptoms. Exclusion criteria in this study were as follows: (1) diagnosis of other eating disorders, (2) recurrent use of inappropriate compensatory behavior, and (3) reported the presence of any illness, or the use of any pharmacological treatment, that might influence eating behavior, body weight, or that would not allow a 12-h fast. Eventually, 116 eligible females agreed to participate: 58 participants were in the BE group and 58 participants were in the control group, and the BE group was matched with the control group by weights. Each group was assigned to hunger or satiety condition randomly. Finally, there were four groups: hungry BED (*N* = 29), satiated BED (*N* = 29), hungry control (*N* = 29), and satiated control (*N* = 29) (Table 1). The study protocol was approved by an Institutional Review Board of Chung-Ang University, Seoul, Republic of Korea (no. 1041078-201910-HRSB-320-01).

**Table 1:** Demographic and clinical characteristics of each group.

	**BE group**		**Control group**		
**Measure**	**Hungry condition (*****N** =* **29)**	**Satiated condition (*****N** =* **29)**	**Hungry condition (*****N** =* **29)**	**Satiated condition (*****N** =* **29)**	**Test statistics (F)**
Age (yrs)	21.103 (1.915)	21.276 (1.944)	21.207 (2.144)	21.241 (1.976)	0.404^*^
BMI	21.310 (2.157)	21.456 (2.242)	21.131 (2.001)	21.144 (2.032)	0.154^*^
EDE-Q	115.414 (38.652)	117.483 (32.068)	55.103 (28.821)	52.069 (22.399)	39.706^*^
BDI	12.241 (5.097)	11.207 (4.872)	4.897 (4.821)	5.448 (4.748)	17.659^*^
STAI-T	60.517 (6.733)	59.103 (7.575)	51.897 (6.915)	50.586 (6.339)	15.265^*^
STAI-S	60.690 (8.553)	57.414 (10.287)	49.759 (8.761)	49.483 (8.114)	11.345^*^
Hunger	69.759 (17.870)	15.241 (15.044)	66.000 (18.188)	18.759 (17.492)	85.086^*^

Mean (standard deviation); ^*^p < 0.001; BE group, binge eating group; control group, weight-matched control. Age, years.

BMI, body mass index; EDE-Q, Eating Disorder Examination Questionnaire; BDI, Beck Depression Inventory; STAI-T, State-Trait Anxiety Inventory-Trait; STAI-S, State-Trait Anxiety Inventory-State; HUNGER, The level of hunger measured by visual analog scale.

### 2.2. Measurement

#### 2.2.1. Self-report questionnaires

The Eating Disorder Diagnostic Scale (EDDS) is a 22-item self-report scale based on DSM criteria for anorexia nervosa, bulimia nervosa, and binge eating disorder (Stice et al., 2000). The Korean version of the EDDS (K-EDDS) was used (Bang et al., 2018b). It was used to identify BE participants and rule out an eating disorder among those in the control group. An overall symptom is calculated from the sum of scores for the first 18 EDDS items. In this study, the Cronbach's α was 0.807.

The Eating Disorder Examination Questionnaire (EDE-Q) is a 36-item self-report measure that assesses the presence and severity of eating disorder psychopathology (Fairburn and Beglin, 1994). The Korean version of EDE-Q version 6.0 was used (Bang et al., 2018a). It consists of a global score and four subscales: eating concern scale, restraint scale, shape concern scale, and weight concern scale. In this study, Cronbach's α was 0.938.

The Beck Depression Inventory (BDI) is a 21-item questionnaire that was originally developed for use with the clinical population, assessing the presence and severity of depression symptoms (Beck et al., 1988). The validated Korean version of BDI was used (Lee et al., 1995). The scale is used to assess the cognitive, emotional, and somatic symptoms of depression. Each item has four choices that describe the severities of each symptom, respectively. Participants choose one option they think to be closest to the state during the past week. In this study, Cronbach's α was 0.821.

The State-Trait Anxiety Inventory (STAI; Spielberger et al., 1970) is used to trait anxiety and state anxiety. The trait version (STAI-T) measures the trait of anxiety, while the state version (STAI-S) measures the state of anxiety. The Korean version of STAI was used (Hahn et al., 1996). The total scores of each subscale are from 20 to 80. The STAI includes 20 items, with greater scores indicating more severe anxiety. In this study, Cronbach's α was 0.834 for STAI-T and 0.750 for STAI-S.

To measure the level of hunger and satiety, the visual analog scale (VAS) ranging from 0 to 100 mm was used. The VAS items consist of a question with “not at all” to “very much”. Participants responded with their own levels of hunger and satiety. Furthermore, to measure the level of wanting and liking, applying the incentive salience model for the rewarding value of food, VAS ranging from 0 to 100 mm was used. The VAS items consist of a question with “not at all” to “very much”. The question to determine wanting was “How much do you want to eat this item right now?”. Liking was determined to the question “How much do you like this item, not considering if you want to eat it right now?” (Stevenson et al., 2017).

The body mass index (BMI) was used to measure participants' physical information. BMI was calculated by dividing weight (kg) by height (m^2^). It is an index that reflects the total amount of body fat. Weight was measured in kilograms, and height was measured in meters using height and weight measuring tools available in the laboratory.

#### 2.2.2. Free-viewing task

Eye-movement data were collected using an eye tracker (Tobii TX300, Tobii Technology AB, Danderyd, Sweden). There were three types of stimuli: high-calorie food, low-calorie food, and non-food cues. Each stimuli type consists of nine images. The high-calorie food cues were items that contained a large amount of fats and sugar, such as hamburgers, ice creams, and chocolates. The low-calorie food cues contained various types of vegetables and fruits. The neutral stimuli included some stationery and household objects. High- and low-calorie cues were determined based on the actual and perceived calories of specific foods, as rated, and standardized in the FATIS (Seo et al., 2020). The FATIS is a database of pictures with normed ratings on addictive images including food, alcohol, nicotine, and non-addictive neutral items. The pair of stimuli was matched using an inspection with respect to complexity, shape, color, brightness, and viewing distance of food cues. Totally, 27 pairs were made (high-calorie food vs. non-food, low-calorie food vs. non-food, high-calorie food vs. low-calorie food). Each pair was presented in a counterbalanced order, and cues were presented twice over on the left and right side of the monitor, conducted 54 trials (Kim et al., 2016). Each pair of cues was presented at a size of 80 × 100 mm with their centers 200 mm apart. Followed by a pair of pictures for 4,000 ms, each trial began with a fixation for 1,000 ms. The eye movements of participants were recorded by an eye-tracking system during the free-viewing task. The eye-tracking data were measured at 120 Hz. All participants performed the free-viewing task in a lighted room, and the size of the monitor was 23 inch with a distance of 60~75 cm between the eyes and monitor. The eye-tracking equipment was calibrated for participants by presenting the five moving dots on the screen, and then the pairs of cues were presented. The software (Tobii TX300, Tobii Technology AB, Danderyd, Sweden) provided a variety of gaze information, involving initial fixation latency score, initial fixation duration score, and gaze duration score.

### 2.3. Procedures

**Figure 1:**
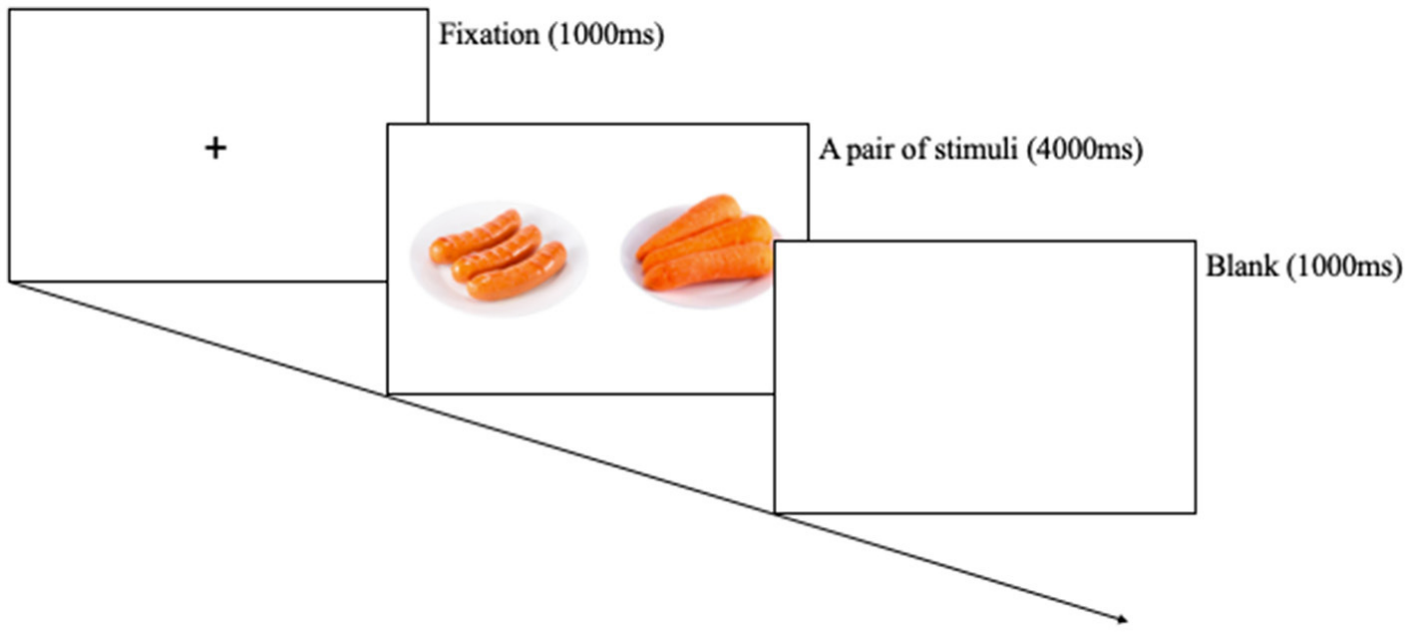
Procedure for the free-viewing task.

Participants were asked not to consume any food, except water, for approximately 12 h prior to the start of the experiment. Upon arrival, participants were provided with information regarding their rights and the procedure. The experiment was scheduled between 8:00 and 10:00 a.m. to align participants' fasting and satiety states as closely as possible. Ultimately, all participants visited the laboratory before 10:00 a.m., and the manipulation checks for the fasting state were based on participants' self-reported responses. When participants arrived at the laboratory, they received instructions on the approved consent form from the Institutional Review Board and voluntarily signed the consent form. Then, participants were randomly assigned to either the hunger or satiety condition, matched for age and body mass index. For the satiety condition, a standard meal was provided at the laboratory to standardize satiety levels. The standard meal consisted of a “gimbap”, approximately 350 kcal, which is a meal consisting of rice, radish, carrots, spinach, and other vegetables wrapped in seaweed. This was done to control participants' satiety levels. All participants in the satiety condition completed hunger and satiety visual analog scales (VASs) before and after the meal to assess their hunger and satiety levels. Participants in the hunger condition completed the hunger VAS only once. Afterward, participants were asked to complete the free-viewing task (Figure 1). All participants were instructed to freely view the computer monitor while minimizing movement during the task. The task consisted of a total of 54 trials. Following the task, participants completed self-report questionnaires. Finally, all participants were provided with a debriefing regarding the experiment. The experimental procedure took approximately 40 min, and all participants received a monetary reward of 10,000 Korean won (approximately 10 USD).

### 2.4. Data analyses

The required sample size for this study was calculated using G*Power 3.1.9.4 (University of Dusseldorf, Dusseldorf, Germany), with an alpha error probability of 0.05 and a power of 0.95. A large effect size of 0.40 was expected with the current sample size. For data analysis, a one-way analysis of variance (ANOVA) was conducted to analyze the differences in the characteristics among the hungry BE, satiated BE, hungry control, and satiated control groups. To examine the differences in attentional bias pattern, three dependent measures were derived from eye-movement data: initial fixation latency, initial fixation duration, and gaze duration. Each score of eye movement data was calculated as the difference between the attentional bias score for high- and low-calorie food cues, and high-calorie food cues and neutral cues. In addition, based on the analysis of the basic characteristics between groups, significant differences were found in the levels of depression and anxiety among the groups. To account for these differences, depression and anxiety levels were set as covariates, and subsequent analyses were conducted. Hypothesis-driven analyses of attentional bias scores were conducted using Group 2 (BE, Control) x Condition 2 (hunger, satiety) two-way analysis of covariance (ANCOVA). Moreover, Group 2 (BE, Control) x Condition 2 (hunger, satiety) x Cue type (high calorie, low calorie) three-way ANCOVA was conducted on self-report wanting and liking VAS. All statistical analyses were conducted using IBM SPSS version 25.0 for Windows, v. 11.0.

## 3. Results

### 3.1. Sample characteristics

A total of 116 participants participated in this study: 29 in the hungry BE group, 29 in the satiated BE group, 29 in the hungry control group, and 29 in the satiated control group. Table 1 shows the group characteristics of the participants analyzed in this study. According to the criteria of matching, there were no significant differences in the mean age [*F*_(3, 112)_ = 0.40, *p* = *0.750*] and the mean BMI [*F*_(3, 112)_ = 0.15, *p* = *0.927*] between the groups. However, there were significant effects of the group for EDE-Q [*F*_(3, 112)_ = 39.71, *p* = *0.0001*, η^2^ = *0.515*], BDI [*F*_(3, 112)_ = 17.70, *p* = *0.0001*, η^2^ = *0.321*], STAI-T [*F*_(3, 112)_ = 15.27, *p* = *0.0001*, η^2^ = 0.290], and STAI-S [*F*_(3, 112)_ = 11.35, *p* = *0.0001*, η^2^ = *0.233*]. Two BE groups had significantly higher eating disorder symptoms, depression, trait anxiety, and state anxiety than did the other two groups. As expected, hungry BE and control groups showed higher hunger than satiated BE and control groups [*F*_(3, 112)_ = 85.09, *p* = *0.0001*, η^2^ = *0.695*], indicating that manipulation was appropriate.

### 3.2. Manipulation check

Table 2 shows the subjective hunger rating before and after consuming a standardized meal. There was no statistically significant interaction between the group and the meal [*F*_(1, 56)_ = 0.59, *p* = *0.446*, η^2^ = *0.01*], and the main effect on the group [*F*_(1, 56)_ = 2.37, *p* = *0.129*, η^2^ = *0.04*]. It is suggested that there was no difference in the hunger level between BE and control groups. Subjective hunger rating using VAS showed a statistically significant main effect of the meal [*F*_(1, 56)_ = 156.35, *p* = *0.0001*, η^2^ = *0.736*], indicating that both BE and control groups showed higher level of hunger before consuming a standardized meal than after the meal.

**Table 2:** Subjective hunger rating before and after consuming a standardized meal.

	**Satiated BE (*N =* 29)**		**Satiated control (N = 29)**		
**Measure**	**Before meal**	**After meal**	**Before meal**	**After meal**	***Test statistics (F)***
Hunger	55.552 (24.606)	15.241 (15.044)	64.345 (21.729)	18.759 (17.492)	0.590

Mean (standard deviation). BE group, binge eating group; control group, weight-matched control group.

### 3.3. Free-viewing task

To examine attentional bias toward food cues, three eye movement scores, involving initial fixation latency score, initial fixation duration score, and gaze duration score, were analyzed for two pairs of cues. The analysis accounted for the potential influence of depressive and anxiety levels (BDI, STAI-T, and STAI-S) by controlling for them as covariates during the analysis, considering that they could be emotional states that can affect attention processes (Smith et al., 2020). Each score of eye-tracking data was calculated as the difference between the score for high- and low-calorie food cues, and high-calorie food cues and neutral cues. Group (BE, control) x Condition (hunger, satiety) two-way ANCOVA was conducted. Furthermore, if there were significant interaction effects, *post hoc* analyses were conducted, and degrees of freedom were adjusted using the Greenhouse–Geisser epsilon to correct for violations of the assumption of sphericity.

#### 3.3.1. Attentional bias toward high-calorie food cues vs. low-calorie food cues among the groups

To examine whether each group exhibits an attentional bias toward high-calorie food cues compared to low-calorie food cues under the hunger condition, the initial fixation latency and initial fixation duration of each group were analyzed. Table 3 shows the mean and standard deviation values of attentional bias toward high-calorie cues vs. low-calorie cues among the groups. First, for the initial fixation latency score, there was no significant interaction between the group and condition [*F*_(1, 109)_ = 0.36, *p* = 0.549, *n.s*.]. Moreover, there was no significant main effect on the group [*F*_(1, 109)_ = 1.81, *p* = 0.182, *n.s*.] and the condition [*F*_(1, 109)_ = 0.90, *p* = 0.345*, n.s*.]. The results indicated that both BE and control groups did not detect high-calorie food cues more quickly than they did the low-calorie food cues, regardless of hunger and satiety.

**Table 3:** Comparison of attentional bias toward high-calorie cues vs. low-calorie cues among groups.

	**BE group**		**Control group**		
**Measure**	**Hungry condition (*****N** =* **29)**	**Satiated condition (*****N** =* **29)**	**Hungry Condition (*****N** =* **29)**	**Satiated Condition (*****N** =* **29)**	**Test statistics (F)**
Initial fixation latency (ms)	−128.569 (152.115)	−84.903 (171.463)	−118.178 (124.776)	−115.250 (120.016)	0.362
Initial fixation duration (ms)	61.465 (81.323)	68.504 (108.823)	64.625 (107.625)	−9.816 (77.947)	5.267^*^
Gaze duration (ms)	632.452 (466.479)	424.906 (414.676)	311.595 (441.079)	255.397 (491.451)	0.739

Mean (standard deviation); In milliseconds (ms) ^*^p < 0.05. BE group, binge eating group; control group, weight-matched control group.

**Figure 2:**
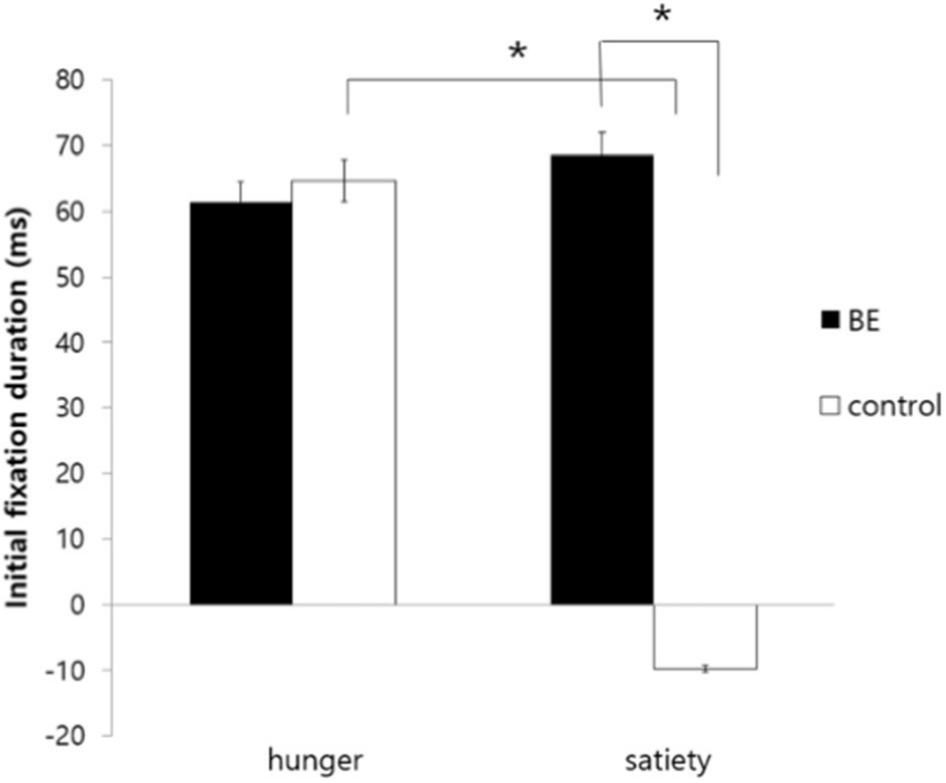
Comparison of initial fixation duration toward high-calorie food cues vs. low-calorie food cues among groups. BE, binge eating group; Control, weight-matched control group; *p < 0.01, error bar: SE.

Second, for initial fixation duration, there was significant interaction between the group and the condition [*F*_(1, 109)_ = 5.27, *p* = 0.024, η^2^ = 0.046]. To determine the source of the interaction, a simple main effects analysis was performed. As a result, there was no difference between hunger and satiety condition in the BE group [*F*_(1, 53)_ = 0.004, *p* = 0.947, *n.s*.]. In contrast, the control group showed higher initial fixation duration to high-calorie food cues compared to low-calorie food cues in the hungry condition, but they were more likely to look initially at low-calorie food in satiated condition [*F*_(1, 53)_ = 9.22, *p* = *0.004*, η^2^ = *0.048*]. Moreover, there was no difference between the BE group and control group in hunger condition [*F*_(1, 53)_ = 1.79, *p* = 0.186*, n.s*.], but the BE group showed higher initial fixation duration to high-calorie food cues vs. low-calorie food cues than the control group in satiated condition [*F*_(1, 53)_ = 8.15, *p* = 0.006, η^2^ = 0.033]. It is indicated that the BE group showed persistent initial attentional bias toward high-calorie food cues both in the hunger and satiety condition, but the control group showed attentional bias only in the hunger condition. On the other hand, there were no significant effects observed for the condition [*F*_(1, 109)_ = 3.54, *p* = 0.063, η^2^ = *0.031*] and the group [*F*_(1, 109)_ = 0.90, *p* = *0.337, n.s*.] (Figure 2).

**Figure 3:**
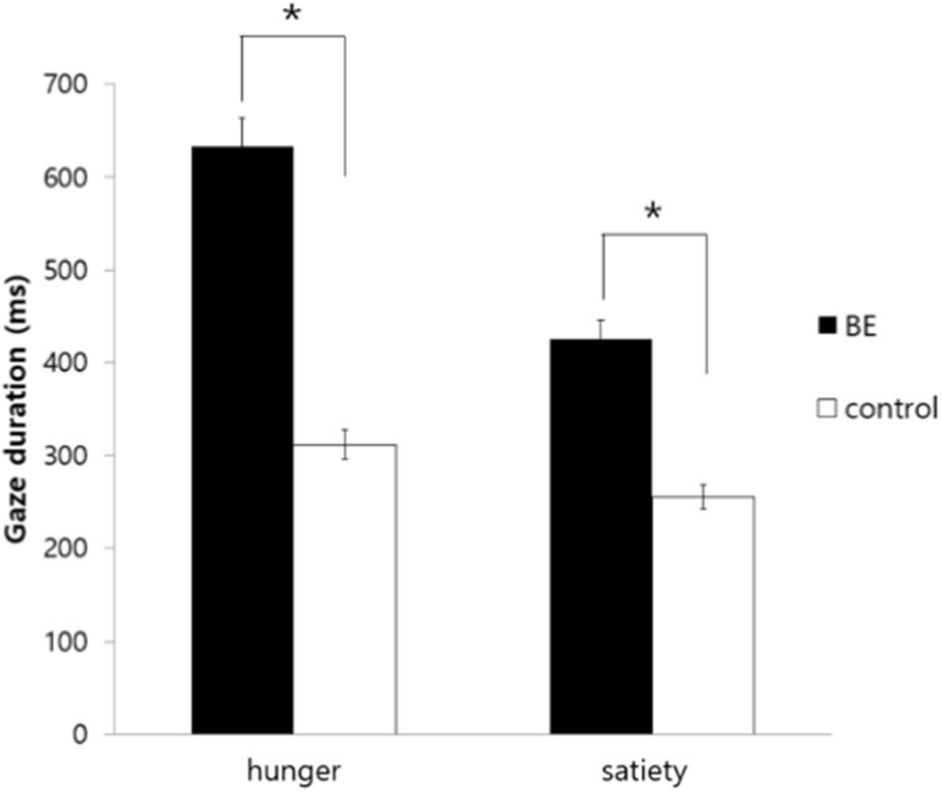
Comparison of gaze duration toward high-calorie food cues vs. low-calorie food cues among groups. BE, binge eating group; Control, weight-matched control group; *p < 0.05, error bar: SE.

To investigate whether participants demonstrating problematic binge eating behaviors under the satiety condition exhibit longer gaze duration toward high-calorie food cues compared to low-calorie food cues, the gaze duration toward food stimuli of each group was analyzed. As a result, there was no significant interaction between the group and the condition [*F*_(1, 109)_ = 0.739, *p* = *0.392, n.s*.] and there was no significant main effect on the condition [*F*_(1, 109)_ = 2.61, *p* = *0.109, n.s*.]. However, there was a significant main effect on the group indicating that the BE group looked at high-calorie food cues longer than low-calorie food cues compared to the control group [*F*_(1, 109)_ = 4.37, *p* = *0.039*, η^2^ = *0.039*] (Figure 3).

#### 3.3.2. Attentional bias toward high-calorie food cues vs. neutral cues among the groups

**Figure 4:**
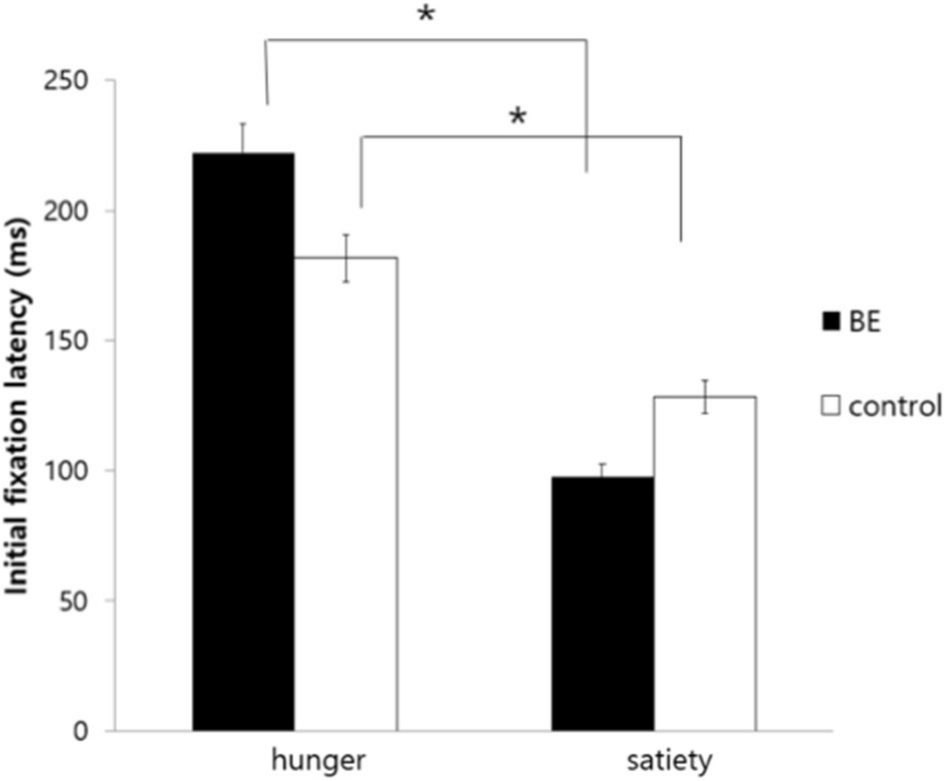
Comparison of initial fixation latency toward high-calorie food cues vs. non-food cues among groups. BE, binge eating group; Control, weight-matched control group; *p < 0.01, error bar: SE.

To examine whether each group exhibits attentional bias toward high-calorie food cues compared to non-food cues under the hunger condition, initial fixation latency, and initial fixation duration of each group were analyzed. Table 4 shows the mean and standard deviation values of attentional bias toward high-calorie cues vs. neutral cues. First, for the initial fixation latency score, there was no significant interaction between the group and the condition [*F*_(1, 109)_ = 0.90, *p* = 0.344*, n.s*.], and the main effect on the group [*F*_(1, 109)_ = 1.81, *p* = 0.182*, n.s*.]. However, there was a significant difference in the condition [*F*_(1, 109)_ = 7.01, *p* = 0.009, η^2^ = 0.060] presenting that all participants showed faster attention engagement in high-calorie food cues when hungry rather than satiated (Figure 4).

**Table 4:** Comparison of attentional bias toward high-calorie food cues vs. non-food cues among groups.

	**BE group**		**Control group**		
**Measure**	**Hungry condition (*****N** =* **29)**	**Satiated condition (*****N** =* **29)**	**Hungry condition (*****N** =* **29)**	**Satiated condition (*****N** =* **29)**	**Test statistics (F)**
Initial fixation latency (ms)	−222.226 (198.515)	−97.433 (180.317)	−181.692 (176.181)	−128.336 (181.347)	0.903
Initial fixation duration (ms)	140.072 (180.618)	98.361 (134.095)	76.754 (163.446)	17.499 (83.797)	0.033
Gaze duration (ms)	1,371.691 (710.136)	854.256 (612.703)	784.701 (827.218)	614.542 (695.017)	1.907

Mean (standard deviation); In milliseconds (ms). BE group, binge eating group; control group, weight-matched control group.

Second, for initial fixation duration, there was no significant interaction between the group and the condition [*F*_(1, 109)_ = 0.03, *p* = 0.855*, n.s*.], and the main effect on the group [*F*_(1, 109)_ = 1.62, *p* = *0.206, n.s*.]. Although not statistically significant, there was a tendency observed for the condition [*F*_(1, 109)_ = 3.19, *p* = 0.077, η^2^ = 0.028], suggesting a cautious implication that participants may have a higher likelihood of viewing high-calorie food cues for a longer duration in the hunger condition compared to the satiety condition.

**Figure 5:**
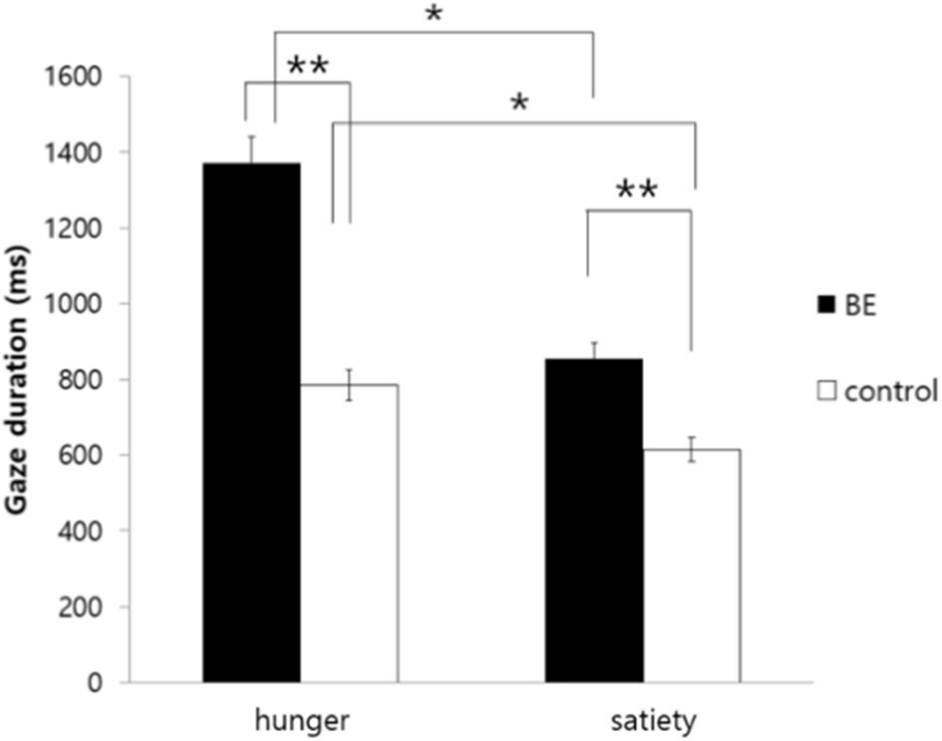
Comparison of gaze duration toward high-calorie food cues vs. non-food cues among groups. BE, binge eating group; Control, weight-matched control group; *p < 0.05; **p < 0.01, error bar: SE.

To investigate whether participants having problematic binge eating behaviors under the satiety condition exhibit longer gaze duration toward high-calorie food cues compared to non-food cues, the gaze duration toward food stimuli of each group was analyzed. As a result, there was no significant interaction between the group and the condition [*F*_(1, 109)_ = 1.91, *p* = 0.170, *n.s*.]. However, there was a significant main effect on the group [*F*_(1, 109)_ = 4.75, *p* = 0.031, η^2^ = 0.042], indicating that the BE group showed attentional bias toward high-calorie food cues vs. neutral cues compared to the control group regardless of hunger and satiety. Moreover, there was a significant main effect on the condition [*F*_(1, 109)_ = 7.06, *p* = 0.009, η^2^ = 0.061], as all hungry participants looked at the high-calorie food cues for a longer time than satiated participants (Figure 5).

### 3.4. Explicit wanting and liking

To assess participants' explicit wanting and liking for high-calorie and low-calorie food cues, their self-reported values on a visual analog scale (VAS) were analyzed. Table 5 shows the mean and standard deviation values for explicit wanting and liking toward high-calorie and low-calorie food cues.

**Table 5:** Comparison of wanting and liking levels toward high-calorie food cues and low-calorie food cues among groups.

	**BE group**		**Control group**		
**Measure**	**Hungry condition (*****N** =* **29)**	**Satiated condition (*****N** =* **29)**	**Hungry condition (*****N** =* **29)**	**Satiated condition (*****N** =* **29)**	**Test statistics (F)**
Wanting_high-calorie	58.395 (21.004)	43.693 (21.621)	47.943 (19.763)	30.395 (18.184)	0.211
Wanting_low-calorie	45.839 (18.115)	37.487 (18.104)	42.969 (18.124)	29.161 (16.327)	0.377
Liking_high-calorie	69.406 (15.958)	65.421 (19.725)	65.441 (17.418)	62.701 (18.205)	0.029
Liking_low-calorie	53.475 (16.635)	54.766 (18.678)	52.812 (14.445)	51.939 (15.376)	0.225

Mean (standard deviation). BE group, binge eating group; control group, weight-matched control group.

Wanting_high-calorie: average of wanting VAS for high-calorie food stimulus, Wanting_low-calorie: average of wanting VAS for low-calorie food stimulus, Liking_high-calorie food cues: average of liking VAS for high-calorie food stimulus, Liking_low-calorie food cues: average of liking VAS for low-calorie food stimulus.

First, in terms of wanting level, the analysis revealed that there was no significant interaction between the group and the condition in wanting for high-calorie food cues [*F*_(1, 109)_ = 0.21, *p* = 0.647, *n.s*.]. There was a main effect on the condition for high-calorie food cues [*F*_(1, 109)_ = 17.01, *p* = *0.0001*, η^2^ = 0.135]. It is indicated that all participants reported higher explicit wanting for high-calorie food when they are hungry rather than satiated. However, there was no significant effect of the group on explicit wanting for high-calorie food cues [*F*_(1, 109)_ = 3.54, *p* = 0.63, *n.s*.]. Furthermore, there was no significant interaction between the group and the condition in wanting low-calorie food cues [*F*_(1, 109)_ = 0.79, *p* = 0.377*, n.s*.]. There was a main effect on the condition [*F*_(1, 109)_ = 12.03, *p* = 0.0008, η^2^ = *0.099*] for low-calorie food cues, indicating that both the BED group and the control group reported higher explicit wanting for low-calorie food cues when hungry than satiated. There was no significant main effect on the group [*F*_(1, 109)_ = 0.44, *p* = 0.510, *n.s*.].

Second, for liking level, there was no significant interaction between the group and the condition in liking both for high-calorie [*F*_(1, 109)_ = 0.03, *p* = 0.865, *n.s*.] and low-calorie food cues [*F*_(1, 109)_ = 0.23, *p* = 0.636, *n.s*.]. There was no significant main effect on the group [*F*_(1, 109)_ = 0.66, *p* = 0.420*, n.s*.] and the condition [*F*_(1, 109)_ = 1.08, *p* = 0.30, *n.s*.] for high-calorie food cues. Moreover, there was no significant main effect on the group [*F*_(1, 109)_ = 0.42, *p* = 0.519, n*.s*.] and the condition [*F*_(1, 109)_ = 0.001, *p* = 0.976, *n.s*.] for low-calorie food cues. It is suggested that there was no difference in liking for high-calorie food cues and low-calorie food cues between the BE group and control group, or between the hunger condition and satiety condition.

## 4. Discussion

This study aimed to examine whether attentional bias for food cues was affected by hunger and satiety maintaining homeostasis in individuals with BE. The result of this study showed that the BE group showed attentional bias toward high-calorie food cues over low-calorie food cues in both hunger and satiety conditions in the early stage of attentional processing. However, the control group showed attentional bias toward high-calorie food cues when hungry, but when satiated they were more likely to look at the low-calorie food cues. In the late stage of attentional processing, the BE group looked at the high-calorie food cues for longer than they did at the low-calorie food cues compared to the control group. Moreover, the BE group reported higher explicit wanting for high-calorie food than the control group did. All participants reported higher explicit wanting for high-calorie food when they are hungry rather than satiated. Finally, there was no difference in explicit liking for the group and the condition.

The main result of this study is that both the BE and control groups showed early attentional bias toward high-calorie food cues over low-calorie food cues in the hunger condition. In the satiety condition, BE participants showed persistent orientation bias toward high-calorie food images, whereas the control group did not. As hypothesized, the effect of the hedonic pathway overriding the homeostatic pathway contributes to the development and maintenance of BE (Novelle and Diéguez, 2018). Normal-weight group showed incentive salience to high-calorie food cues only when hungry according to the homeostasis pathway (Lutter and Nestler, 2009). While it is adaptive to quickly detect and allocate attention toward high-calorie food during energy depletion, it is inappropriate to show the attentional bias toward high-calorie food cues, regardless of the condition in the BE group. In addition, the continuous hyperreaction of high-calorie food cues suggests why the majority of people with BED are overweight or obese (Field et al., 2013).

This result supported the main hypothesis that the reward system activity is abnormally enhanced as exposure to palatable food cues in individuals with binge eating behaviors (Pool et al., 2016). In line with the incentive-sensitization theory, high-calorie food seems to be more salient than low-calorie food in the BE group because it is the reward-related cues. The attentional bias in the early stage of attentional processing presented the automatic engagement in high-calorie food cues, reflecting the implicit motivation to obtain a reward (Fox et al., 2001). As the cues triggered reactivity to high-calorie food cues may be due to conditioning systems, attentional bias limited to high-calorie food cues may be caused by a personal history of binge eating (Berridge and Robinson, 2003). The study examining food selection and intake of overweight women with BED showed that participants with BED consumed a greater percentage of energy as fat and a lesser percentage as protein than did participants without BED during the binge meal (Yanovski et al., 1992). Moreover, it is suggested that palatable food containing sugar and fat, most of which are high-calorie foods, have addictive properties (Gearhardt et al., 2011; Smith and Robbins, 2013). The results may be the evidence of addiction such as the consumption of palatable food in individuals with binge eating behaviors.

The study shows that initial orientation bias toward high-calorie food cues vs. neutral cues appeared in all participants when hungry. The absence of group differences in participants' orientation bias toward high-calorie food cues vs. non-food is consistent with other eye-tracking studies in adults and adolescents who binge eat (Schag et al., 2013; Sperling et al., 2017). However, preferential orientation bias toward food stimuli was found in adults with BE episodes in real scenes (Popien et al., 2015) and in studies using reaction time-based measures (Schmitz et al., 2014, 2015; Sperling et al., 2017). The difference in results might be explained by the use of different experimental procedures and stimulus types. Moreover, another reason why this study did not show any difference between high-calorie food cues and neutral cues may be because of the ceiling effect. The ceiling effect may have occurred, as paying attention to high-calorie food cues is the most important issue for survival when hungry.

As expected, the longer gaze duration for high-calorie food cues compared to low-calorie food cues or neutral food cues in individuals with BE was replicated in our study. There were relatively consistent results that people with BE showed slower disengagement of food cues (Schag et al., 2013; Popien et al., 2015; Schmidt et al., 2016; Sperling et al., 2017). However, like the control group, the BE group also showed longer gaze duration for high-calorie food cues vs. neutral food cues when they were hungry than when they were satiated in this study. The result is different from those of the group with obesity that may be related to the reward system dysregulation (Nijs et al., 2010). As the maintained stage of attention measures more strategic attention (Fox et al., 2001), the BE group can also be affected by hunger and satiety in explicit desire (i.e., explicit wanting). This has something in common with the result of self-reported explicit wanting, applying the classification of explicit and implicit wanting in the incentive-sensitization theory. The BE group reported higher explicit wanting for high-calorie food than the control group did, and all participants reported higher explicit wanting for high-calorie food when they are hungry rather than satiated. The results may suggest that computations of wanting to incorporated the current physiological state in the BE group (Zhang et al., 2009). Another possible explanation is that there may be a risk of binge eating or loss of control when hungry rather than satiated. It is consistent with the result that dietary restraint would predict binge eating episodes (Freeman and Gil, 2004).

When comparing high-calorie cues with low-calorie cues, the late stage of attentional bias toward high-calorie food cues is still evident in the BE group. This supports, to some extent, the approach-avoidance bias presented in previous studies (Schmidt et al., 2016). The results were different from other eating disorders, such as AN and BN (Brooks et al., 2011). For example, the eye-tracking studies showed that AN and BN attended to food cues for a shorter time than the control group did (Blechert et al., 2011). Moreover, the eye-tracking study demonstrated that the bulimic tendency group detected high-calorie food cues faster than neutral food cues initially and avoided attentional maintenance (Kim et al., 2016). People with BE are more similar to people with obesity that showed consistent attentional bias in the early and late stages. While people with obesity tend to allocate their attention to both high-calorie and low-calorie food cues, BE showed attentional bias only for the high-calorie food cues (Nijs et al., 2010). This demonstrated that, unlike people with obesity, intervention focusing on the high-calorie food cues may be required in individuals with binge eating behaviors.

There are several limitations in this study. First, in this study, the participants had a BMI index of approximately 21, which is within the average range for individuals in Korea and indicates that they did not exhibit issues of obesity or overweight. Therefore, we assumed that consuming “gimbap,” which is commonly regarded as a typical Korean meal, would induce a basic level of satiety. Although both the BED group and the control group reported reduced hunger after the meal, individual differences in the degree of satiety may exist, suggesting the possibility that participants did not fully experience satiety. Therefore, in future studies, it would be beneficial to use various physiological and psychological measures (e.g., blood samples), rather than relying solely on self-report evaluations, to assess the participants' level of hunger in a more objective manner. Second, the food images formed a homogeneous category, whereas non-food images depicted items from various categories. Thus, it is possible that more attention may be paid to food stimulus because they were of the same category. However, an overriding consideration in selecting the stimuli was within each picture pair, in which the food and neutral cues were matched as closely as possible for complexity, color, and brightness. In future, it would seem desirable to select non-food images from a single category. Third, this study did not consider food intake to examine BE after an attentional bias toward high-calorie food cues. In a future study, having a bogus taste test could provide additional evidence of BE.

To conclude, the current study offers a suggestion that high-calorie food perception is biased in individuals with binge eating behaviors vs. weight-matched female controls. This is the first evidence to examine the differences in attentional patterns between BE and weight-matched control groups in the consideration of demands of the internal milieu based on the incentive-sensitization theory. As visual food cues are particularly prominent in society, understanding the cognitive process of exposure to visual food cues in BE is of great importance in developing potential behavioral therapies, environmental alterations, and public health measures. Moreover, based on the results, attention bias modification could be implemented to modify the specific attentional bias to high-calorie food cues.

## Data availability statement

The original contributions presented in the study are included in the article/supplementary material, further inquiries can be directed to the corresponding author.

## Ethics statement

The studies involving human participants were reviewed and approved by Chung-Ang University IRB. The patients/participants provided their written informed consent to participate in this study.

## Author contributions

J-MW: conceptualization, methodology, validation, formal analysis, investigation, data curation, writing, and visualization. J-HL: conceptualization, methodology, validation, resources, supervision, project administration, and funding acquisition. G-EL: secondary writing, visualization, and modified the submitted version. All authors contributed to the article and approved the submitted version.
